# CT Angiography for the Diagnosis of Hemorrhagic Jejunal Diverticulosis: A Rare Cause of Obscure GI Bleeding

**DOI:** 10.7759/cureus.107484

**Published:** 2026-04-21

**Authors:** Salma Abouchiba, Wiame Bougrine, Hajar Ouazzani, Ismail Chaouche, Amal Akammar, Nizar El Bouardi, Meriem Haloua, Badreddine Alami, Y. Lamrani, Meryem Boubbou, Mustapha Maaroufi

**Affiliations:** 1 Department of Radiology, Hassan II University Hospital, Sidi Mohamed Ben Abdellah University, Fez, MAR; 2 Department of Mother and Child Radiology, Hassan II University Hospital, Sidi Mohamed Ben Abdellah University, Fez, MAR

**Keywords:** abdominal ct angiography, jejunal diverticulosis, lower gastrointestinal bleeding, obscure gastrointestinal bleeding, radiologic diagnosis, small bowel diverticula, small bowel hemorrhage

## Abstract

Jejunal diverticulosis (JD) is a rare condition of the small intestine characterized by acquired pseudodiverticula arising along the mesenteric border. Although most cases remain asymptomatic, complications may occur in a minority of patients and include diverticulitis, perforation, intestinal obstruction, malabsorption, and, more rarely, GI hemorrhage. Bleeding originating from jejunal diverticula is an uncommon but potentially life-threatening cause of obscure GI bleeding (OGIB). We report the case of a 59-year-old woman who presented to the ED with melena associated with symptomatic anemia. Initial gastroduodenal endoscopy failed to identify a source of bleeding. Subsequently, contrast-enhanced CT angiography of the abdomen was performed and demonstrated multiple jejunal diverticula. One diverticulum contained spontaneous hyperdense material on non-contrast images, compatible with intraluminal blood, and was associated with a dilated submucosal vascular structure, suggesting the site of recent hemorrhage. JD is a rare but important cause of OGIB. When endoscopic investigations are inconclusive, CT angiography plays a crucial role in identifying the bleeding source and guiding management. Awareness of this entity and its imaging features is essential for radiologists and clinicians involved in the evaluation of GI hemorrhage.

## Introduction

Jejunal diverticulosis (JD) is an uncommon condition, with a reported prevalence ranging from 0.3% to 1.3% in radiologic series and up to 2.3% in autopsy studies [1]. Unlike Meckel diverticulum, jejunal diverticula are acquired pseudodiverticula composed of mucosa and submucosa herniating through the muscularis propria at sites of vascular penetration, typically along the mesenteric border [1,2].

Most patients remain asymptomatic; however, approximately 10-30% may develop complications, some of which are potentially life-threatening [2,3]. GI bleeding secondary to JD is particularly rare and often presents as obscure GI bleeding (OGIB), defined as persistent or recurrent bleeding after negative initial upper and lower endoscopic evaluations, largely due to the limited accessibility of the jejunum to conventional endoscopy [4].

In the acute setting, CT angiography plays a central role in diagnosis and management, as it allows rapid, noninvasive detection of active bleeding and precise localization of the source, often guiding therapeutic decision-making when endoscopic evaluation is inconclusive or not feasible. We report a case of hemorrhagic JD diagnosed by CT angiography, highlighting the diagnostic value of this modality in the evaluation of OGIB and emphasizing its role in guiding appropriate management.

## Case presentation

A 59-year-old woman with a history of chronic rheumatologic disease treated intermittently with corticosteroids and NSAIDs and a history of gout without ongoing treatment presented to the ED with melena evolving over two days. The episode was associated with diffuse abdominal discomfort, asthenia, and dizziness, without hematemesis or other digestive symptoms. There was no history of liver disease, cardiovascular disease, diabetes, or chronic kidney disease. She denied alcohol or tobacco use.

On admission, the patient was conscious and hemodynamically stable, with a blood pressure of 116/64 mmHg but tachycardic at 124 beats per minute. She was afebrile (37.5°C) with oxygen saturation of 96% on room air. Clinical examination revealed marked cutaneo-mucosal pallor and conjunctival pallor. Abdominal examination showed a soft, non-distended abdomen with mild epigastric tenderness, without palpable masses or organomegaly. Digital rectal examination confirmed the presence of melena.

Initial laboratory investigations demonstrated severe anemia with a hemoglobin level of 7 g/dL, associated with microcytosis (mean corpuscular volume 78.5 fL), suggestive of iron deficiency anemia in the context of chronic or subacute blood loss. Platelet count was within normal limits (227,000/mm³), and coagulation parameters were normal (prothrombin time, 100%), excluding a coagulopathy-related bleeding disorder. Ferritin level was decreased (16 ng/mL), further supporting iron deficiency. Renal function, electrolytes, and inflammatory markers were unremarkable (Table 1).

**Table 1:** Laboratory findings of our patient on admission

Parameter	Patient value	Normal range
Hemoglobin	7.0 g/dL	12.0-16.0 g/dL
Mean corpuscular volume	78.5 fL	80-100 fL
Mean corpuscular hemoglobin concentration	32.4 g/dL	32-36 g/dL
White blood cells	7,580/mm³	4,000-10,000/mm³
Neutrophils (polymorphonuclear neutrophils)	4,410/mm³	2,000-7,000/mm³
Lymphocytes	2,430/mm³	1,000-4,000/mm³
Platelets	227,000/mm³	150,000-400,000/mm³
Prothrombin time	100%	70-100%
C-reactive protein	2 mg/L	<5 mg/L
Creatinine	6 mg/L	6-12 mg/L
Urea	0.42 g/L	0.15-0.45 g/L
Ferritin	16 ng/mL	30-300 ng/mL

The Glasgow-Blatchford score was calculated at 8, indicating a high risk of clinically significant bleeding. The patient was managed with proton pump inhibitor therapy (intravenous bolus followed by continuous infusion) and blood transfusion, with a target hemoglobin level of 10 g/dL. Urgent esophagogastroduodenoscopy revealed erythematous and atrophic antrofundic gastritis without evidence of active bleeding or high-risk stigmata. Colonoscopy performed during the same hospitalization was unremarkable. Given the persistence of anemia and the absence of an identifiable source on endoscopic evaluation, a contrast-enhanced abdominal CT angiography was performed to investigate OGIB.

Imaging findings

**Figure 1:**
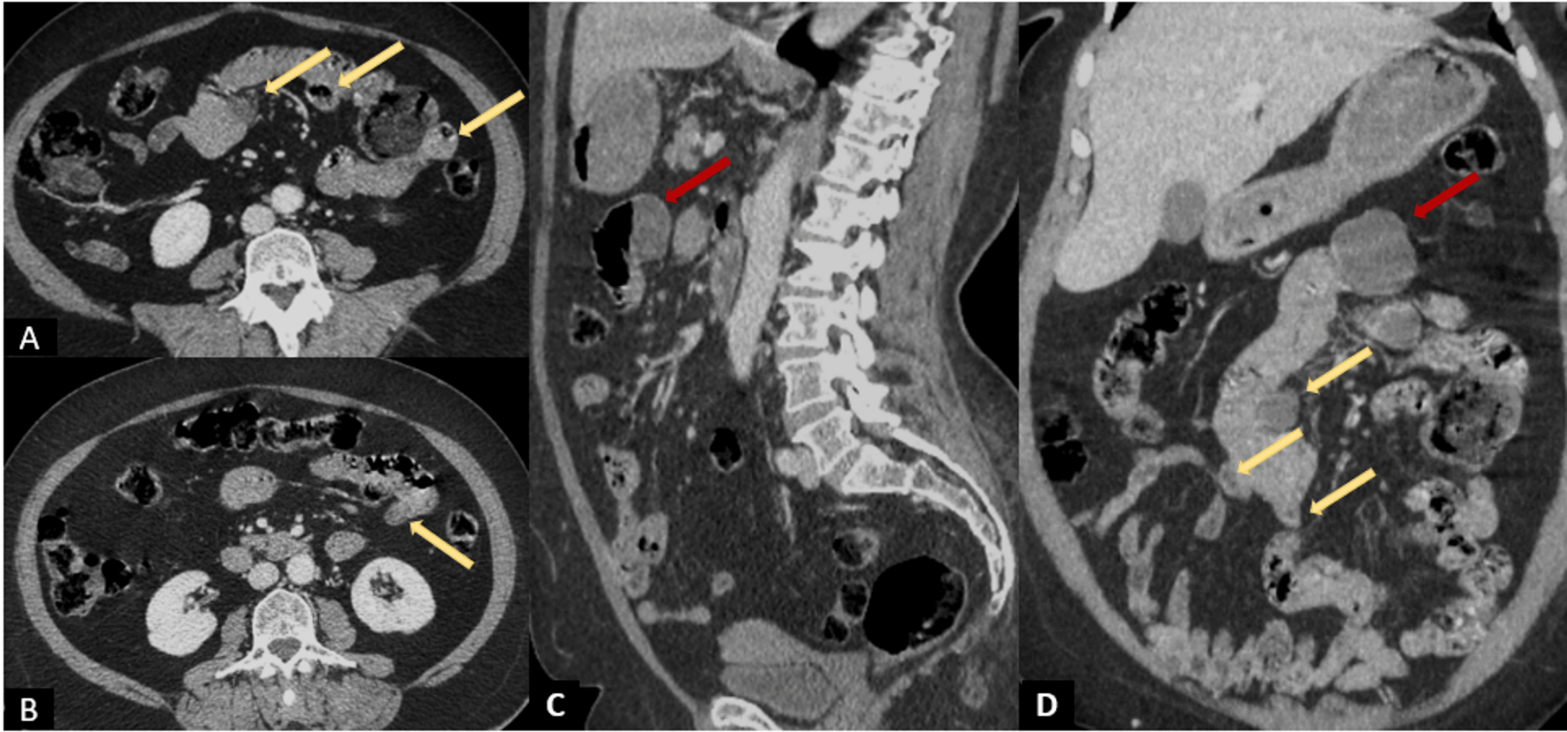
Contrast-enhanced abdominal CT (portal venous phase) with axial (A, B), sagittal (C), and coronal (D) reformations demonstrating multiple air-filled and air-fluid outpouchings arising from the jejunal wall (yellow arrows). The largest lesion is located in the proximal jejunum (red arrows).

CT angiography was performed using a multiphasic protocol, including non-contrast, arterial, and portal venous phases. The examination demonstrated multiple diverticula arising from the jejunum, predominantly along the mesenteric border, consistent with JD (Figure 1). One diverticulum, located in the proximal jejunum, showed spontaneous hyperdense content on non-contrast images, consistent with intraluminal blood or clot.

**Figure 2:**
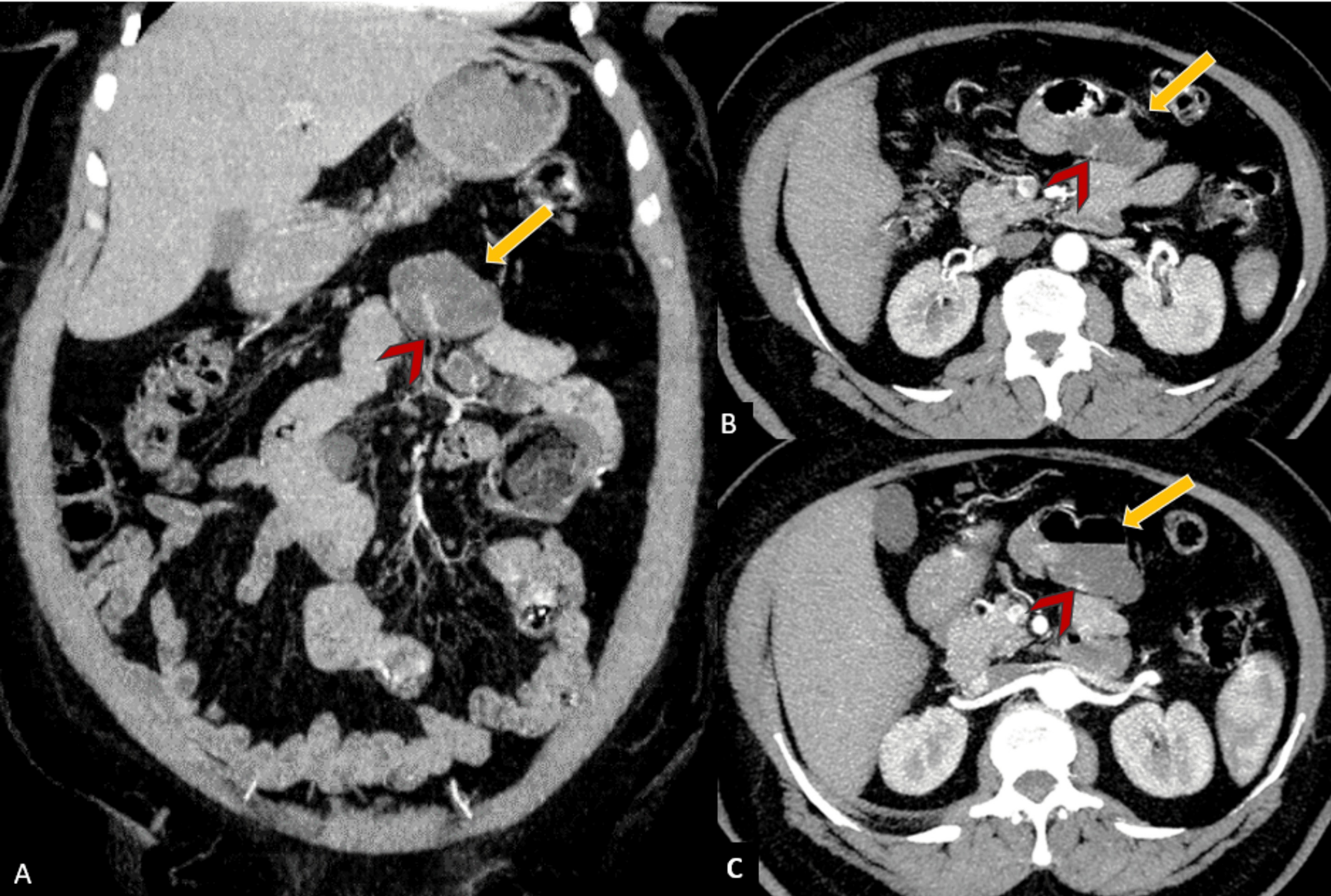
Arterial phase CT angiography with coronal (A) and axial (B, C) images showing prominent dilated vascular structures (red arrowheads) within the posterior wall of the proximal jejunal diverticulum (yellow arrow).

On arterial and portal venous phase images, a dilated submucosal vascular structure was identified adjacent to this diverticulum, suggesting the likely site of recent hemorrhage (Figure 2). No active contrast extravasation was observed at the time of imaging, favoring a recent rather than ongoing bleeding event.

There were no associated signs of diverticulitis, including bowel wall thickening or peridiverticular fat stranding. No evidence of perforation, pneumoperitoneum, bowel obstruction, or mesenteric ischemia was identified. No suspicious masses or lymphadenopathy were detected. Overall, the imaging findings were highly suggestive of hemorrhagic JD as the source of GI bleeding.

Clinical course and outcome

Following supportive management, including blood transfusion and proton pump inhibitor therapy, the patient showed significant clinical improvement with stabilization of hemoglobin levels (8.6 g/dL on follow-up testing). Given this favorable evolution and the absence of hemodynamic instability, a conservative management approach was adopted.

A control esophagogastroduodenoscopy performed during follow-up showed no evidence of an alternative source of upper GI bleeding, further supporting a small bowel origin. No recurrent bleeding was observed during hospitalization. The patient was discharged with oral iron supplementation for correction of iron deficiency (ferritin 16 ng/mL) and scheduled for outpatient follow-up. At the time of discharge, she remained hemodynamically stable with no signs of ongoing or recurrent GI bleeding.

## Discussion

JD is a rare condition of the small intestine, predominantly affecting elderly patients and characterized by acquired pseudodiverticula arising along the mesenteric border [1,2]. These diverticula result from mucosal and submucosal herniation through weak points of the muscular layer at sites of vascular penetration [2,3]. Although most cases remain asymptomatic, approximately 10-30% of patients develop complications, some of which may be severe and potentially life-threatening [1,4]. The spectrum of complications associated with JD is broad and includes diverticulitis, perforation, intestinal obstruction, hemorrhage, abscess formation, and, more rarely, volvulus or fistulization [1,5]. These complications often present with nonspecific clinical features, which contributes to diagnostic delay and increased morbidity.

Diverticulitis and perforation

Diverticulitis represents the most frequently reported complication of JD and typically manifests with abdominal pain, fever, and inflammatory syndrome [4,6]. CT is the imaging modality of choice, demonstrating findings such as focal bowel wall thickening, mesenteric fat stranding, and sometimes localized fluid collections [6,7]. In more advanced cases, diverticulitis may progress to perforation. This may be localized, resulting in abscess formation, or generalized, leading to peritonitis requiring urgent surgical intervention [1,4]. Early diagnosis using cross-sectional imaging is essential, as clinical presentation may mimic other acute abdominal conditions.

Intestinal obstruction and other complications

Intestinal obstruction is another recognized complication and may occur due to adhesions, volvulus, intussusception, or enterolith formation within diverticula [6,8]. CT imaging plays a central role in identifying the mechanism and level of obstruction. Chronic complications include bacterial overgrowth resulting from stasis within diverticula, which may lead to malabsorption and nutritional deficiencies [2]. Although less common, abscess formation and volvulus have also been described in complicated cases [6,9].

Hemorrhagic complications

GI bleeding is among the rarest but most clinically significant complications of JD [10]. Although uncommon, it may present as acute and sometimes severe hemorrhage, leading to significant anemia or hemodynamic compromise. The pathophysiology of hemorrhage is thought to involve erosion or rupture of submucosal vessels located within the diverticulum, similar to the mechanism described in colonic diverticular bleeding [2,10]. Additional contributing factors may include mucosal inflammation and vascular ectasia. Clinically, bleeding often presents as melena or hematochezia and may be intermittent, which contributes to diagnostic difficulty. Standard upper and lower endoscopic examinations frequently fail to identify the bleeding source due to the anatomical inaccessibility of the jejunum [10].

Role of imaging

In cases of negative endoscopic evaluation, imaging plays a crucial role. CT angiography has emerged as a key modality for the evaluation of OGIB, allowing both anatomical and vascular assessment [5]. Typical CT findings include hyperdense intraluminal content on non-contrast images, active contrast extravasation in cases of ongoing bleeding, and identification of abnormal or dilated vascular structures adjacent to diverticula [5]. Even in the absence of active bleeding, CT may reveal indirect signs of recent hemorrhage, as demonstrated in our case.

Management

Management of hemorrhagic JD depends on the clinical presentation and severity of complications. As in the case of our patient, conservative treatment, including fluid resuscitation and transfusion, may be sufficient in cases of self-limited bleeding [7,10]. In cases of persistent or recurrent hemorrhage, interventional radiology procedures such as selective arterial embolization may be required, while surgical resection remains the definitive treatment in refractory cases [11].

## Conclusions

JD is a rare but important cause of OGIB, particularly when initial endoscopic investigations are negative. Hemorrhagic complications, although uncommon, can be clinically significant and require a high index of suspicion. CT angiography plays a crucial role in identifying the bleeding source and detecting subtle signs of recent hemorrhage, even in the absence of active extravasation. This case highlights the educational value of recognizing this uncommon etiology and its imaging features, emphasizing the importance of CT angiography in guiding timely and appropriate management while avoiding diagnostic delays.
